# Investigating the impact of cannabis

**DOI:** 10.7554/eLife.94760

**Published:** 2023-12-20

**Authors:** Merrick Pierson Smela

**Affiliations:** 1 Harvard University Cambridge United States

**Keywords:** metabolism, cannabis, primordial germ cells, development, Mouse

## Abstract

The psychoactive component of cannabis, ∆9-THC, affects cell growth and metabolism in early embryonic cell types in mice.

**Related research article** Verdikt R, Armstrong AA, Cheng J, Yang X, Allard P. 2023. Metabolic memory of Δ9-Tetrahydrocannabinol exposure in pluripotent stem cells and primordial germ cells-like cells. *eLife* **12**:RP88795. doi: 10.7554/eLife.88795.

Cannabis is the most widely used illicit drug in the world. Its consumption has also increased in pregnant women. In the United States alone, around 12% of pregnant women are reported to use cannabis at least once per month during the first trimester (Volkow et al., 2019).

The psychoactive component of cannabis is ∆9-tetrahydrocannabinol (∆9-THC), which activates cannabinoid receptors in the brain, including the receptor CB1. Previous research has shown that CB1 is already expressed in the early mouse embryo (Wang et al., 2003), but so far it has been unclear if ∆9-THC affects the development of mice. Now, in eLife, Patrick Allard from the University of California Los Angeles and colleagues – including Roxane Verdikt as first author – report new findings that help to answer this question (Verdikt et al., 2023).

To study the effect of ∆9-THC on development, Verdikt et al. used embryonic cells of mice that had been cultured in the laboratory to resemble different developmental stages. Embryonic stem cells were extracted from the inner cell mass of early mouse embryos. These cells are similar to the cells in an embryo before implantation in the uterus. When treated with relevant signaling factors, these cells can develop into epiblast-like cells, which are similar to mouse embryo cells shortly after implantation in the uterus. Treatment with different signaling factors causes epiblast-like cells to develop into primordial germ cell-like cells, which can eventually become eggs and sperm (Hayashi et al., 2011). Embryonic stem cells and epiblast-like cells are two kinds of pluripotent stem cells, meaning they have unlimited potential to self-renew and to differentiate into mature cells that make up an embryo (Dierolf et al., 2021; Hayashi et al., 2011). However, embryonic stem cells are in a naïve pluripotent state, corresponding to an earlier developmental stage than epiblast-like cells, which are in a primed pluripotent state (Nichols and Smith, 2009). The switch from naïve to primed pluripotency is accompanied by a metabolic shift in energy production. It changes from a process called oxidative phosphorylation, which reduces oxygen to generate energy, to glycolysis, which splits sugar molecules to generate energy (Dierolf et al., 2021; Tsogtbaatar et al., 2020).

Verdikt et al. found that exposing the embryonic stem cells to ∆9-THC increased their growth rate at doses as low as 1 nanomolar (Figure 1). This is much lower than the concentration found in recreational light cannabis users, which varies between 22–58 nanomolar for cannabis containing low ∆9-THC levels (Pacifici et al., 2020). However, the growth rate of epiblast-like cells was unaffected by ∆9-THC treatment, even at higher doses. In comparison, human embryonic stem cells, which are in a primed pluripotent state and thus closer to epiblast-like cells than mouse embryonic stem cells, displayed a slightly decreased growth rate when exposed to 100 nanomolar of ∆9-THC for six days.

**Figure 1.**
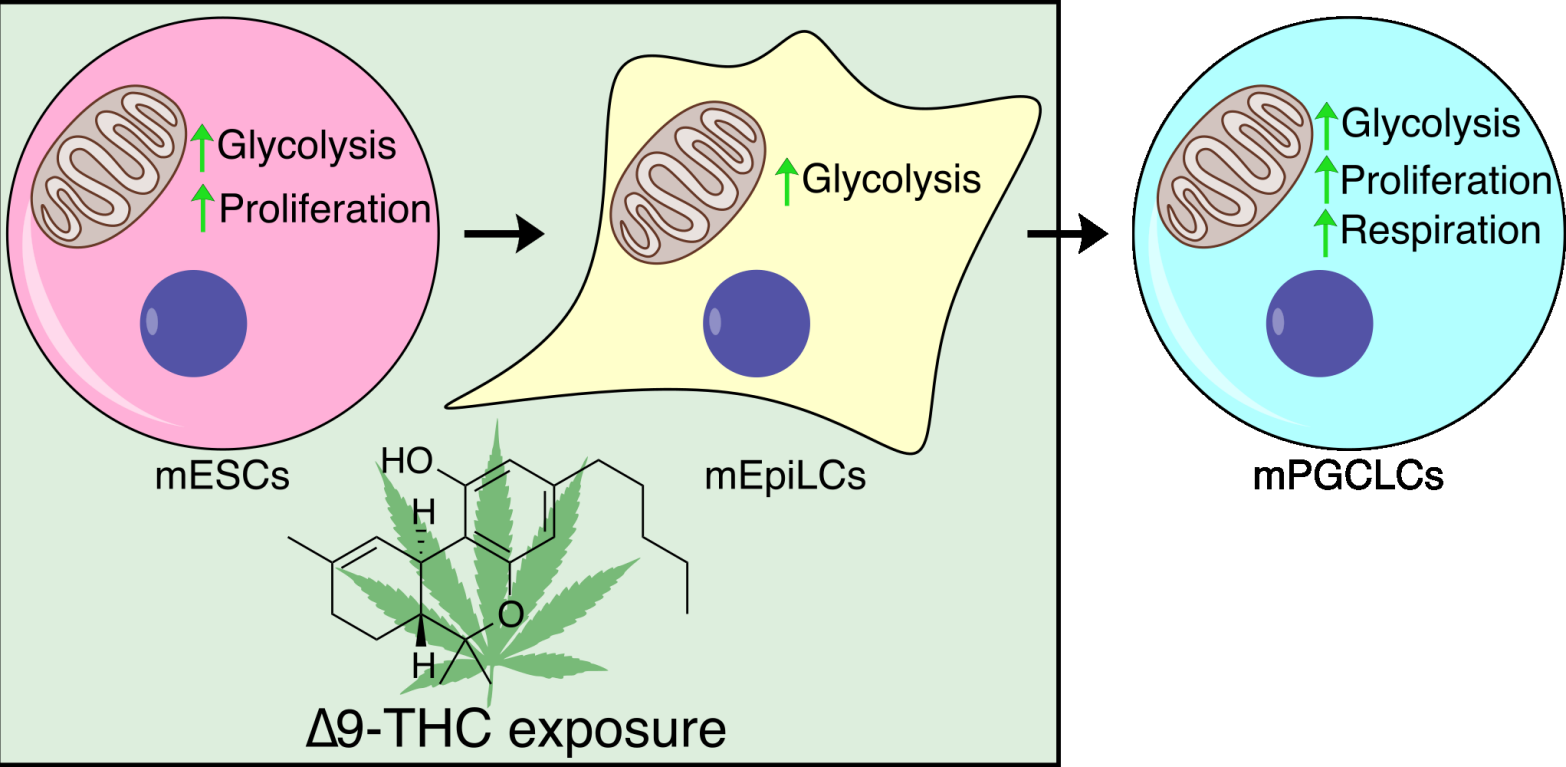
The effect of cannabis on embryonic stem cell types from mice. The psychoactive component of cannabis, ∆9-THC, alters the growth and metabolism of early embryonic cell types of mice. Treatment with ∆9-THC increased glycolysis in mouse embryonic stem cells (mESCs, pink circle) and epiblast-like cells (mEpiLCs, yellow shape). It also increased the proliferation rate of the mESCs, but not mEpiLCs. Primordial germ cell-like cells (mPGCLCs, blue circle) derived from the ∆9-THC-treated cells also showed increased rates of glycolysis, respiration and proliferation, even in the absence of ongoing ∆9-THC exposure.

To identify why ∆9-THC affects the growth of mouse embryonic stem cells and epiblast-like cells differently, Verdikt et al. studied the role of the cannabinoid receptor CB1 more closely. The researchers found that CB1 was expressed on the surface of both cell types. But treatment with rimonabant, a drug that inhibits CB1 signaling, only blocked the pro-growth influence of ∆9-THC in the embryonic stem cells, indicating that CB1 is responsible for these effects.

To find out why ∆9-THC does not impact epiblast-like cells, which is clearly not due to a lack of CB1 protein, Verdikt et al. looked at the metabolism rates of the different cells. This revealed that ∆9-THC increased the rate of glycolysis in both embryonic stem cells and epiblast-like cells. The embryonic stem cells used the glycolysis products to build new biomolecules and to increase their rate of growth, and this effect was blocked following treatment with a glycolysis inhibitor. In epiblast-like cells, however, the increased rate of glycolysis did not affect their growth rate. This could be due to the already higher glycolysis turnover prior to ∆9-THC exposure observed in these cells.

Verdikt et al. then studied the cumulative effect ∆9-THC on cell development. They started treatment with ∆9-THC either during the embryonic stem cell phase or the epiblast-like cells stage, and then induced the epiblast-like cells to further differentiate into primordial germ cell-like cells, which are the earliest precursors of the egg and sperm. Exposure to ∆9-THC during either cell stage increased cell growth in the primordial germ cell-like cells. The primordial germ cell-like cells derived from the ∆9-THC-treated group also showed changes to metabolism and gene expression even after ∆9-THC treatment had stopped, suggesting that a ‘metabolic memory’ can be passed on to cells in the next developmental stage.

This study raises three important questions. First, what is the role of CB1 in the early embryo? It has been shown that certain molecules that bind to CB1, such as anandamide, regulate the implantation process of an embryo under normal conditions (Wang et al., 2003). The fact that blocking CB1 in the embryonic stem cells of mice decreased their growth rate suggests that this receptor has some basal activity in these cells. Second, do the results on primordial germ cell-like cells also apply to their human counterpart? Human and mouse primordial germ-like cells differ in many ways (Kobayashi et al., 2017; Kojima et al., 2017). Verdikt et al. did not find an increased growth rate in human embryonic stem cells, and it could be expected that ∆9-THC may affect human cells differently. Third, how long does the apparent metabolic memory last? As primordial germ cell-like cells can develop all the way to eggs and sperm, early ∆9-THC exposure could potentially affect the fertility of the offspring if this memory remains (Hayashi et al., 2011; Hikabe et al., 2016).

Overall, the study by Verdikt et al. suggests that physiologically relevant doses of ∆9-THC have metabolic effects on embryonic stem cells in mice, and that these effects can persist during the differentiation of germ cells. If similar effects exist in humans, this would be significant for public health.

## References

[bib1] Dierolf JG, Watson AJ, Betts DH (2021). Differential localization patterns of pyruvate kinase isoforms in murine naïve, formative, and primed pluripotent states. Experimental Cell Research.

[bib2] Hayashi K, Ohta H, Kurimoto K, Aramaki S, Saitou M (2011). Reconstitution of the mouse germ cell specification pathway in culture by pluripotent stem cells. Cell.

[bib3] Hikabe O, Hamazaki N, Nagamatsu G, Obata Y, Hirao Y, Hamada N, Shimamoto S, Imamura T, Nakashima K, Saitou M, Hayashi K (2016). Reconstitution in vitro of the entire cycle of the mouse female germ line. Nature.

[bib4] Kobayashi T, Zhang H, Tang WWC, Irie N, Withey S, Klisch D, Sybirna A, Dietmann S, Contreras DA, Webb R, Allegrucci C, Alberio R, Surani MA (2017). Principles of early human development and germ cell program from conserved model systems. Nature.

[bib5] Kojima Y, Sasaki K, Yokobayashi S, Sakai Y, Nakamura T, Yabuta Y, Nakaki F, Nagaoka S, Woltjen K, Hotta A, Yamamoto T, Saitou M (2017). Evolutionarily distinctive transcriptional and signaling programs drive human germ cell lineage specification from pluripotent stem cells. Cell Stem Cell.

[bib6] Nichols J, Smith A (2009). Naive and primed pluripotent states. Cell Stem Cell.

[bib7] Pacifici R, Pichini S, Pellegrini M, Rotolo MC, Giorgetti R, Tagliabracci A, Busardò FP, Huestis MA (2020). THC and CBD concentrations in blood, oral fluid and urine following a single and repeated administration of “light cannabis.”. Clinical Chemistry and Laboratory Medicine.

[bib8] Tsogtbaatar E, Landin C, Minter-Dykhouse K, Folmes CDL (2020). Energy metabolism regulates stem cell pluripotency. Frontiers in Cell and Developmental Biology.

[bib9] Verdikt R, Armstrong AA, Cheng J, Yang X, Allard P (2023). Metabolic memory of Δ9-Tetrahydrocannabinol exposure in pluripotent stem cells and primordial germ cells-like cells. eLife.

[bib10] Volkow ND, Han B, Compton WM, McCance-Katz EF (2019). Self-reported medical and nonmedical cannabis use among pregnant women in the United States. JAMA.

[bib11] Wang H, Matsumoto H, Guo Y, Paria BC, Roberts RL, Dey SK (2003). Differential G protein-coupled cannabinoid receptor signaling by anandamide directs blastocyst activation for implantation. PNAS.

